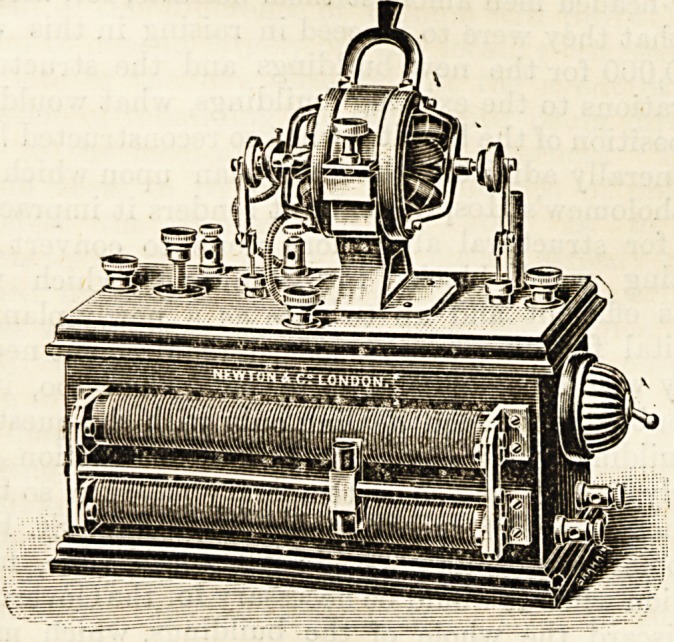# New Appliances and Things Medical

**Published:** 1903-01-10

**Authors:** 


### NEW APPLIANCES AND THINGS MEDICAL.

### ROENTGEN RAY WORK WITH THE ALTERNATING CURRENT.

Mr. Russell S. Wright has contrived an apparatus for utilising alternating currents for Roentgen ray work, which he describes as follows: To convert the alternating current into continuous for the benefit of *x*-ray and other workers is a problem that has up to the present defied complete solution, and many so-called "rectifiers" have been introduced with, at any rate, only very partial success. One very important fact, however, has frequently been lost sight of, or, at least, has not been sufficiently attended to.

For *x*-ray work, or, in fact, for induction coil work of any sort, the current required is *not* continuous, but rather pulsating or intermittent in character, and even where the original supply is continuous, a "make and break" of some description is necessary in order to provide such an intermittent current. Now, fortunately, the alternating supply *does* adapt itself, readily and easily, to provide the necessary intermittent, but uni-directional, current, and the illustration shows an instrument designed by the writer for obtaining such a result.

Briefly, the method is to employ a "make and break" similar to those frequently used with the continuous current, but so arranged that during the periods of "make,"

the current passing shall be entirely in one direction, while all the reverse pulsations, *i.e.*, the periods during which the current is passing in the reverse direction, shall be entirely covered by the periods of "break." The instrument is really a motor-driven mercury contact breaker, working in series with the coil, the motor being driven by a parallel circuit of the same alternating current. A sliding resistance provided (shown in the front in the illustration) is to adjust the speed of the motor until it runs in synchronism with the pulsations of the alternating circuit.

The ordinary pattern of synchronous motor was not very suitable for the purpose as these motors are very difficult to start. The one employed was, therefore, specially designed, a touch with the hand when the current is switched on being sufficient to start it, and synchronism, when the resistance is adjusted, being almost instantly obtained. The synchronous pace can readily be detected, (1) by the even "hum" or "buzz" made by the motor when running synchronously, and (2) by the almost total absence of sparking at the commutator brushes.

Another very simple means for detecting synchronism was suggested by Mr. F. H. Glew, at whose laboratory in Clapham Road the experimental work in connection with the instrument was done. A small ivory peg is screwed to the end of the armature spindle and revolves with it. Viewed by the light of an ordinary incandescent lamp supplied by the same alternating current, this peg is apparently seen to slowly revolve, the movement becoming slower as the speed of the motor approaches synchronism, and appearing as a stationary arm the moment true synchronism is obtained. Once adjusted, the resistance need not be touched again unless the current should greatly vary, when a slight movement of the slide again produces synchronism.

The strength of the current passing through the coil can be regulated by the handle at the top of the motor. This handle is to revolve the entire motor (which can be done while the instrument is running, and without upsetting the synchronous pace in any way), and is to "phase" the periods of make and break, the greatest effect from the coil being obtained when all the "makes" occur at zero potential, and all the "breaks" when the successive pulsations of the current in the one direction are at their maximum strength. By moving this handle the coil, working with, say, 100 volts, alternating current can be made to give any result from an almost infinitesimal discharge up to a torrent of sparks of the full length that the coil is capable of producing.

By turning a small brass milled head a wooden block can be lowered into the mercury in order to bring the level of this to the right height. When once the instrument is correctly adjusted it is impossible to tell by the appearance of the *x*-ray tube that the coil is not being worked by the continuous current, and it has been used by the writer, at the Roentgen Society's conversazione last March for nearly four hours almost without intermission and without any readjustment being made. As will any ordinary mercury contact breaker, the mercury needs cleaning and renewing at times, and also the insulating liquid with which it is covered; the latter may be either methylated spirit or paraffin oil, or even (though with some loss of efficiency) water.

Owing to the peculiar construction of the motor the instrument can be used as an ordinary mercury contact breaker for a coil working with the continuous current, the motor again being driven by the house supply and giving good results worked in this way. It has also been used successfully by Mr. Glew as a rectifier for charging accumulators from the alternating supply, though as only one-half the current is employed, the operation is rather a lengthy one.

## Figures and Tables

**Figure f1:**